# A Young COVID-19-Positive Male Patient Presented With Bilateral Pulmonary Emboli and Multiple Strokes

**DOI:** 10.7759/cureus.14722

**Published:** 2021-04-27

**Authors:** Islam Younes, Zamir Singh, Walaa Hammad, Ahmed Mowafy, Carlos Remolina

**Affiliations:** 1 Internal Medicine, Rutgers New Jersey Medical School/Trinitas Regional Medical Center, Elizabeth, USA; 2 Internal Medicine, St. George's University School of Medicine, Elizabeth, USA; 3 Pathology and Laboratory Medicine, Ain Shams University Hospital, Cairo, EGY; 4 Pulmonary and Critical Care Medicine, Rutgers New Jersey Medical School/Trinitas Regional Medical Center, Elizabeth, USA

**Keywords:** covid 19, pulmonary embolism (pe), deep vein thrombosis (dvt), stroke

## Abstract

COVID-19 has been repeatedly related to a variety of extra-pulmonary manifestations since its emergence. COVID-19-positive patients have been shown to develop neurological deficits, deep venous thrombosis, acute kidney injury, acute hepatic injury, and myocarditis, among other conditions.

The mechanism of some of these injuries remains unclear, but one factor that has been revealed is hypercoagulability. A hypercoagulable state, whether secondary to dysfunctional coagulation cascades or microvascular angiopathy, has been reported in the literature in COVID-19 patients. We present a case of a patient diagnosed with COVID-19 presented with venous thromboembolism and then shortly developed innumerable strokes.

## Introduction

Coronavirus (severe acute respiratory syndrome coronavirus 2 [SARS-CoV-2]), that began in 2019 and has been named COVID-19, is associated with a wide clinical presentation range. Clinical presentation ranges from being asymptomatic or having mild symptoms to severe respiratory symptoms as well as extra-respiratory symptoms.

Prothrombotic complications including arterial and venous thromboembolism are frequently reported complications in COVID-19-positive patients. The exact mechanism is unknown; however, different mechanisms are proposed [1,2]. Stroke has also been reported in COVID-19 patients, and this was found to be associated with high morbidity and mortality [3].

We report a case of a 47-year-old male patient diagnosed with COVID-19 presented with venous thromboembolism and then shortly developed innumerable strokes with no evidence of intracardiac shunt.

## Case presentation

**Figure 1:**
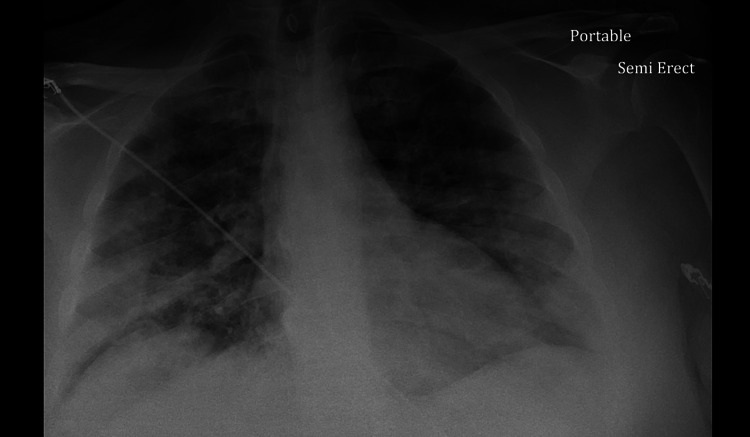
Chest x-ray showing bilateral patchy opacities.

**Figure 2:**
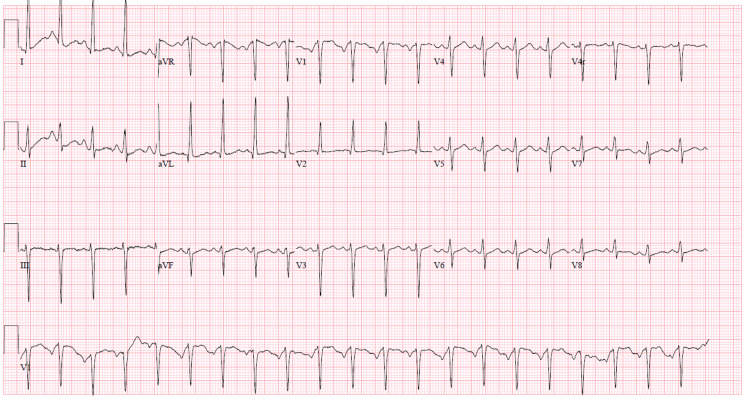
EKG showing sinus tachycardia. EKG, Electrocardiogram.

**Figure 3:**
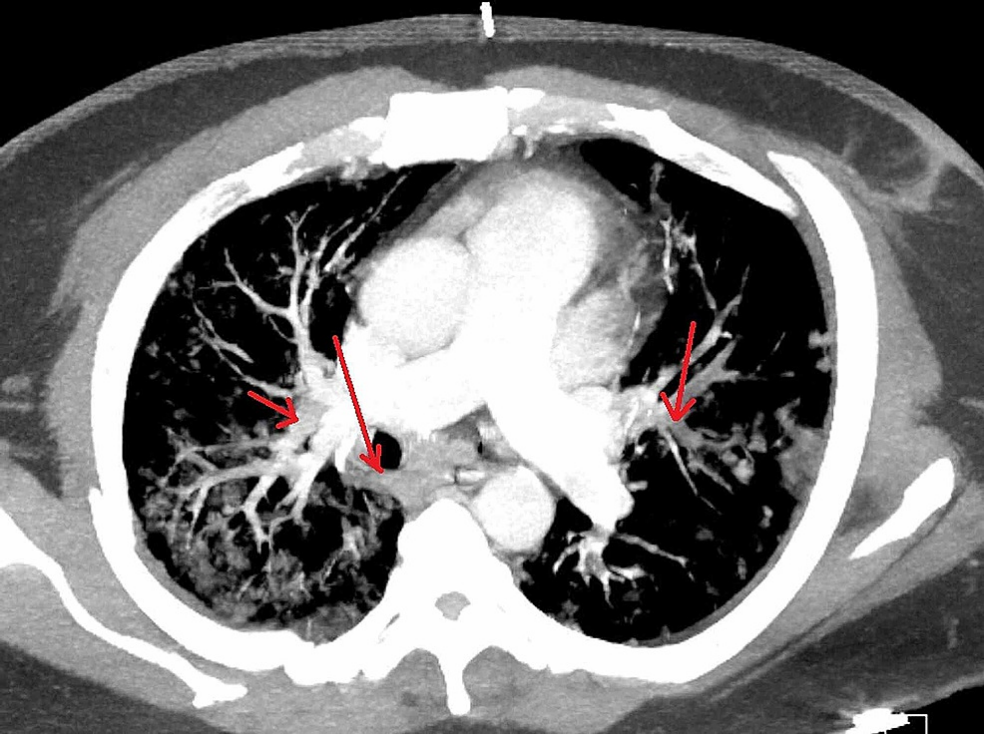
CT chest angiography showing ground-glass opacities and bilateral subsegmental pulmonary embolisms (red arrows).

**Figure 4:**
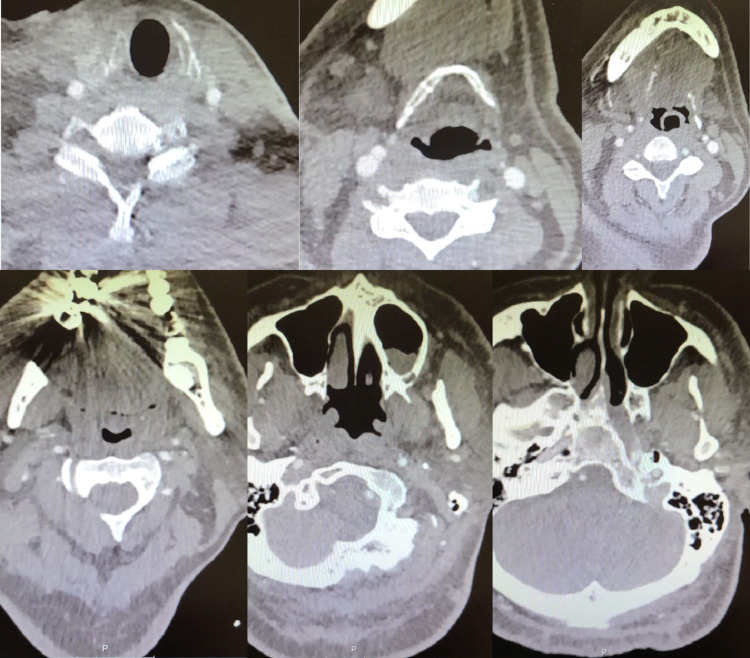
CT angiography of neck and head showing no evidence of hemodynamically significant vertebral arteries stenosis.

**Figure 5:**
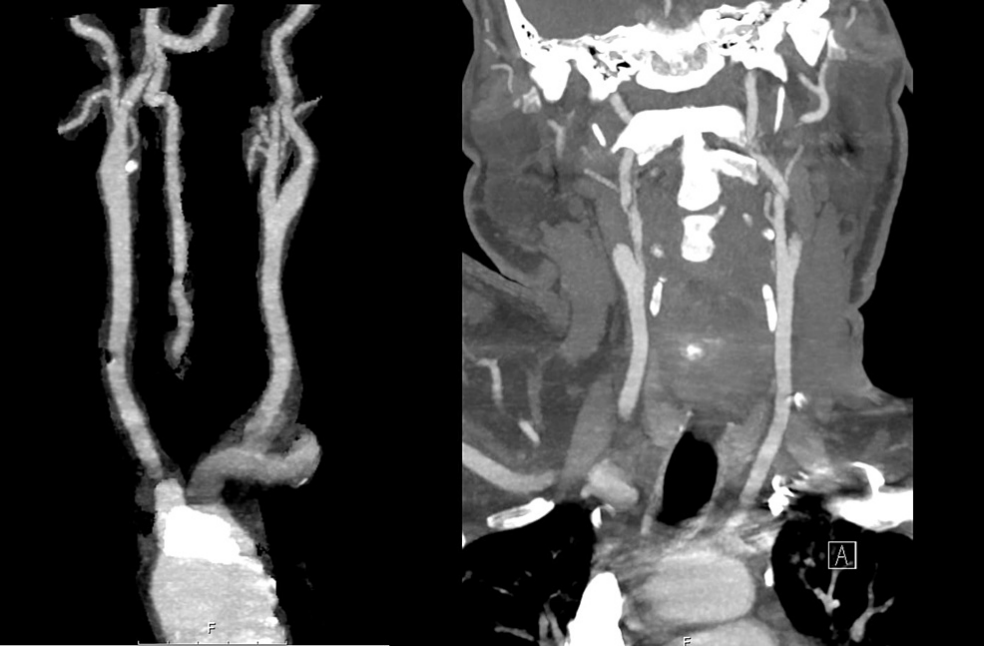
CT angiography of neck showing no evidence of hemodynamically significant carotid artery stenosis.

**Figure 6:**
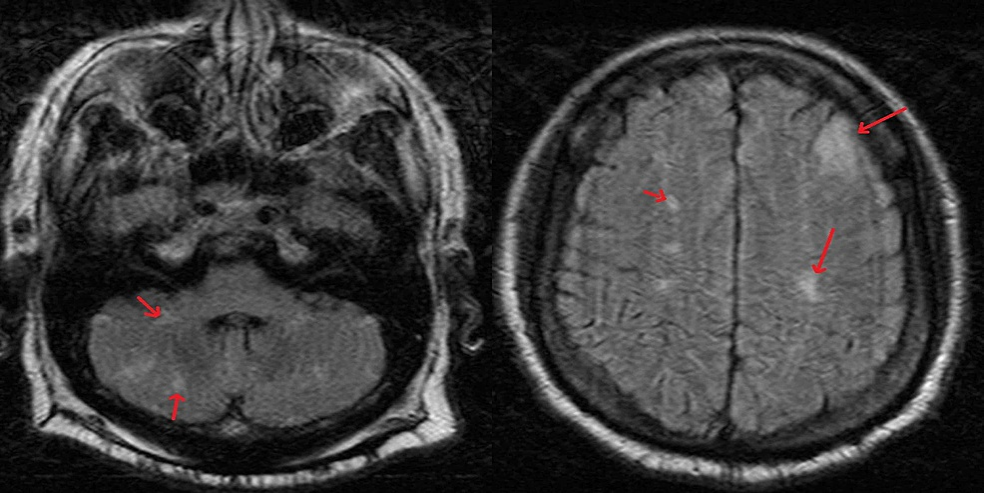
MRI of brain showing acute/subacute bilateral cerebellar hemispheres (left side) and left frontal lobe (right side). MRI, Magnetic resonance imaging.

 47-year-old male patient with a past medical history of hypertension, diabetes mellitus type 2, relapsing-remitting, non-active multiple sclerosis presented to the emergency department (ED) with a three-day history of worsening exertional shortness of breath associated with pleuritic chest pain and productive cough. The patient’s vitals showed a fever of 38.3°C, tachycardia with heart rate (HR) 133, tachypnea with respiratory rate (RR) 25, BP 150/80, and oxygen saturation of 80% on room air. Physical exam was remarkable for bilateral basal crackles. Chest x-ray (CXR) showed extensive patchy peripheral and central parenchymal opacities (Figure 1). Electrocardiogram (EKG) showed sinus tachycardia (Figure 2). Labs were remarkable for highly elevated D-dimer > 5,000 ng/ml [0-230 ng/ml] with mild leukocytosis 14.4 K/UL [4.8-10.8 K/UL], troponin 0.1 ng/ml [0-0.4 ng/mL], and brain natriuretic peptide (BNP) 50 pg/ml [< 100 pg/ml]. Computed tomography angiography (CTA) of the chest showed bilateral multifocal ground-glass opacity with left lower segmental and bilateral subsegmental pulmonary emboli (Figure 3). Rapid COVID-19 polymerase chain reaction (PCR) came back positive. The patient was admitted to the intensive care unit (ICU) and started on oxygen supplementation by nasal cannula, heparin infusion, dexamethasone 6 mg daily, and remdesivir. Echocardiography showed left ventricular ejection fraction (LVEF) of 25%-30% with no evidence of right heart strain; however, the patient was not clinically in heart failure. Lower extremities venous duplex showed bilateral distal and proximal deep venous thrombosis up to the popliteal veins level. On day three after admission, the patient was found to be confused with new neurologic deficits including ataxia and dysmetria. CT of head showed moderate size acute/subacute left frontal lobe infarct with suspected small bilateral subacute cerebellar infarcts with no evidence of intracerebral hemorrhage (ICH). Neurologist was consulted who advised no tissue plasminogen activator (tPA) and continuation of his current medical therapy. CT angiography of head and neck did not show evidence of vascular stenosis (Figures 4, 5). MRI of brain showed innumerable acute and subacute infarctions including small acute/subacute infarcts of the bilateral cerebellar hemispheres, bilateral caudate nuclei, and moderate to large acute/subacute infarct of the left frontal lobe (Figure 6). A repeat echo with contrast and bubble study showed no evidence of mural thrombi or intracardiac shunts (Videos 1, 2). The patient remained stable on medical therapy with an improvement of his neurological findings and was downgraded to the telemetry unit, and then discharged to subacute rehabilitation on goal-directed medical therapy with planning for cardiac angiography as an outpatient to rule out ischemic cardiomyopathy.

**Video 1:** Echocardiography with bubble study does not show evidence of intracardiac shunt.

**Video 2:** Echocardiography with contrast showing no evidence of left ventricular mural thrombus.

## Discussion

Thrombotic events have emerged as complications of severe acute respiratory syndrome coronavirus 2 (SARS-CoV-2). These include myocardial infarctions, acute cerebrovascular events, venous thromboembolism (VTE), disseminated intravascular coagulation (DIC), and acute limb ischemia among others. These complications have been increasingly seen during the disease process in patients with COVID-19. Given the serious nature of these events, it is essential to analyze their occurrence in order to establish preventative measures and determine management protocols in hospitalized patients. This can ensure the reduction of morbidity and mortality associated with the disease.

The thrombogenic properties of COVID-19 are thought to stem from a hyperinflammatory response to the virus and its role in altering enzyme activity. In a similar mechanism to SARS-CoV-1, COVID-19 has been shown to interact with angiotensin-converting enzyme 2 (ACE-2), which is involved in the conversion of angiotensin 1 to angiotensin 2. However, COVID-19 has been found to bind to this enzyme with a higher affinity than that of SARS-CoV-1 and down-regulating the enzyme just like SARS-CoV-1 [4]. This prevents the conversion of angiotensin 1, which is involved in creating a proinflammatory state along with processes such as vasoconstriction [4]. This proinflammatory state has been associated with the development of a prothrombotic state. This is demonstrated by the fact that elevated levels of certain cytokines, such as Il-6, IL-8, C-reactive protein (CRP), and tumor necrosis factor-alpha (TNA-alpha), are associated with an increased risk for thrombotic events such as venous thromboembolism (VTE) [5]. In addition to this, the inflammatory process activates endothelial cells and platelets, both of which are involved in the coagulation cascade that leads to the development of thrombi.

The association between thrombogenic events and COVID-19 is compounded in patients who are already at an increased risk for these events. Included in this at-risk group are hospitalized patients, who are notably at an increased risk of VTE. In fact, over half of the hospitalized patients are seen to be at risk for VTE, with rates in the absence of prophylaxis ranging from 10% to 80% [6]. Additional risk factors include ICU, patients with cancer, obesity, pregnancy, a history of stroke, VTE, or myocardial infarction (MI) among others. Given the additional risk present in these patient groups, it is prudent that clinicians recognize this and factor it into their management plans for COVID-19 patients.

In ischemic strokes, the arterial occlusion is generally embolic in nature. It is either atheroembolic due to atherosclerotic disease in large vessels, such as the carotid or vertebral arteries, or cardioembolic due to valvular heart disease or atrial fibrillation [7]. In our patient, cardiogenic causes were ruled out, with an echocardiogram confirming an absence of ventricular thrombi, valvular disease, or a patent foramen ovale. The most likely source of the ischemic stroke was the microthrombi originating in the intracranial vasculature itself. The intrinsic small-vessel disease has been found to be responsible for approximately a quarter of ischemic strokes with limited understanding of the pathogenesis, given that these arteries are too small for good visualization with imaging during the stroke [7]. This small-vessel disease was most likely exacerbated by the inflammatory and prothrombotic states induced by a COVID-19 infection, thus leading to ischemic stroke in the patient.

When considering patients at risk for stroke in the setting of a COVID-19 infection, it is important to identify the vascular risk factors that may predispose them to develop cerebrovascular accidents. The most common risk factors for the development of a stroke include hypertension, dyslipidemia, diabetes mellitus, atrial fibrillation, coronary artery disease, smoking, previous stroke, heart failure, alcoholism, pacemakers, and malignancies [8]. Thus, with COVID-19 patients presenting with these risk factors, it is essential that further steps be taken to minimize stroke risk. Educating the responsible physicians and establishing measures to ensure the identification of these risk factors will certainly aid in reducing the incidence of stroke in patients with COVID-19. It is also essential that healthcare professionals working with COVID-19 patients are alerted to the specific associated conditions that present with this disease. Given that the risk of a disabling stroke even after a minor stroke or transient ischemic attack is front-end loaded [7], early identification of stroke in a patient can aid in reducing the morbidity and mortality associated with both stroke and COVID-19.

## Conclusions

COVID-19 has been associated with thromboembolic disease in critically and non-critically ill patients. Further studies are needed for a better understanding of the pathophysiology and the appropriate anticoagulant prophylactic and therapeutic regimens in COVID-19 patients.
